# *Chlamydomonas* as a model system to study cilia and flagella using genetics, biochemistry, and microscopy

**DOI:** 10.3389/fcell.2024.1412641

**Published:** 2024-05-30

**Authors:** Wallace F. Marshall

**Affiliations:** Department Biochemistry and Biophysics, University of California San Francisco, San Francisco, CA, United States

**Keywords:** ciliogenesis, intraflagellar transport, flagellar length control, axoneme, motility

## Abstract

The unicellular green alga, *Chlamydomonas reinhardti*i, has played a central role in discovering much of what is currently known about the composition, assembly, and function of cilia and flagella. *Chlamydomonas* combines excellent genetics, such as the ability to grow cells as haploids or diploids and to perform tetrad analysis, with an unparalleled ability to detach and isolate flagella in a single step without cell lysis. The combination of genetics and biochemistry that is possible in *Chlamydomonas* has allowed many of the key components of the cilium to be identified by looking for proteins that are missing in a defined mutant. Few if any other model organisms allow such a seamless combination of genetic and biochemical approaches. Other major advantages of *Chlamydomonas* compared to other systems include the ability to induce flagella to regenerate in a highly synchronous manner, allowing the kinetics of flagellar growth to be measured, and the ability of *Chlamydomonas* flagella to adhere to glass coverslips allowing Intraflagellar Transport to be easily imaged inside the flagella of living cells, with quantitative precision and single-molecule resolution. These advantages continue to work in favor of *Chlamydomonas* as a model system going forward, and are now augmented by extensive genomic resources, a knockout strain collection, and efficient CRISPR gene editing. While *Chlamydomonas* has obvious limitations for studying ciliary functions related to animal development or organ physiology, when it comes to studying the fundamental biology of cilia and flagella, *Chlamydomonas* is simply unmatched in terms of speed, efficiency, cost, and the variety of approaches that can be brought to bear on a question.

## 1 Introduction

Cilia and flagella are complex organelles with diverse functions and highly conserved molecular structures, found throughout the eukaryotic tree of life. While a number of different model organisms can be used to study the assembly and function of cilia, the unicellular green alga *Chlamydomonas reinhardtii*, sometimes known as “green yeast,” stands out as the most tractable genetic and biochemical model organism, which also has specific advantages for live cell imaging and quantitative analysis. The central premise of this article is that, if you want to study the general mechanisms of how cilia assemble and function, as opposed to the specific roles that they play in a developmental or disease context, *Chlamydomonas* is the most convenient and powerful system in which to carry out such studies. As a haploid, unicellular organism, that grows on plates or liquid culture with a doubling time of a few hours, *Chlamydomonas* allows rapid, high-throughput, yeast-like approaches that make it the most convenient and efficient model organism for genetic analysis of cilia. As a system in which cells can be grown in biochemical quantities in a matter of days with minimal equipment and inexpensive media, and in which flagella can be cleanly detached from the cell body without cell lysis, *Chlamydomonas* is by far the best system for biochemical analysis of cilia. *Chlamydomonas* is also ideal for live-cell imaging of flagella by TIRF microscopy. All of these advantages will be discussed in detail below.

Another “advantage” is not intrinsic to the cell but a product of its history, and that is the long legacy of genetic analysis that has resulted in a vast array of mutations, all available in the *Chlamydomonas* Genetics Center, which any researcher can use for their own new purposes. In this sense, *Chlamydomonas* occupies a similar place in the genetic analysis of cilia and flagella as *Drosophila* does in the genetic analysis of animal development. But because of so much interest in *Chlamydomonas* genetics, many modern tools also exist. A high quality genome is available with excellent annotation, knockout collections exist so that for almost any gene one can obtain mutants simply by pulling the appropriate plates out of the freezer, and CRISPR gene editing allows insertion of tags at endogenous loci and other such genomic manipulations.

Historically, *Chlamydomonas* played a leading role in many of the key discoveries about cilia and flagella, for example, the discovery of intraflagellar transport (Kozminski et al., 1993; Cole et al., 1998), which led to the explosion of interest in ciliary diseases when it was found that a protein component of the *Chlamydomonas* IFT system corresponded to the Tg737 mouse mutation that caused polycystic kidney disease (Pazour et al., 2000). Indeed, the vast majority of ciliary proteins appear to be conserved between *Chlamydomonas* and humans, with differences being limited mainly to proteins involved in developmental signaling or specific physiological sensing functions that a free living cell would not need to have. For an excellent overview of the history of *Chlamydomonas* flagellar research, see (Rosenbaum, 2009). Here we will focus on what one can do now, using *Chlamydomonas*; its unique advantages compared to virtually any other model organism; and the new types of high-throughput and quantitative studies that it enables.

## 2 Cell biology of *Chlamydomonas* flagella

### 2.1 Anatomy of the *Chlamydomonas* cell

**FIGURE 1:**
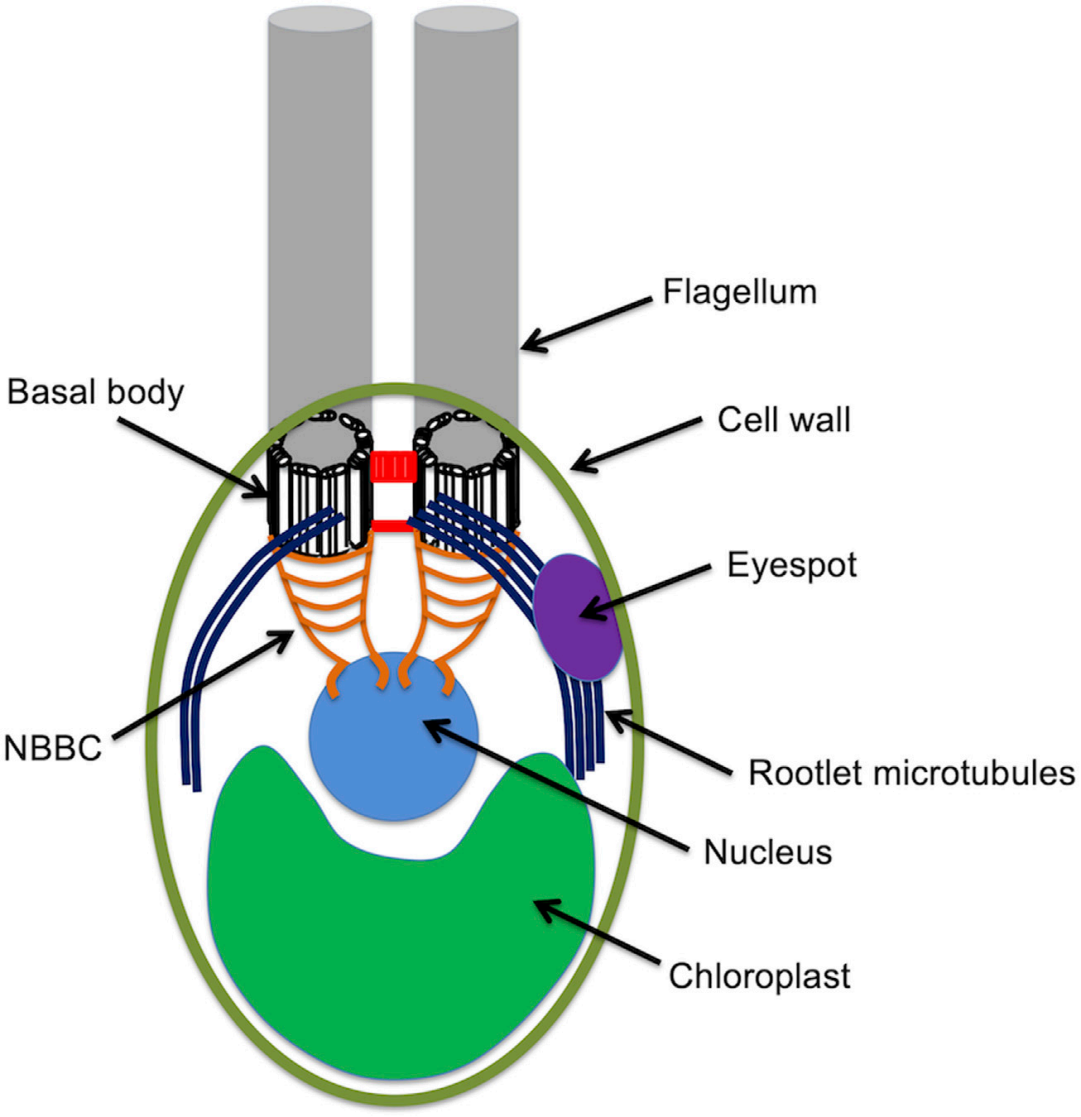
The anatomy of the Chlamydomonas cell.

*Chlamydomonas* cells are spheroidal, around 10 microns in diameter, with two conspicuous flagella protruding from one end, conventionally called the anterior end of the cell. The cell is highly polarized, with virtually all internal structures occupying defined positions relative to the anterior-posterior axis (Figure 1). Just under the flagella are the basal bodies from which the flagella extend. Associated with these basal bodies are a complex arrangement of fibers that connect the basal bodies to each other, to the intracellular microtubule cytoskeleton, and to the nucleus. The nucleus sites below (posterior to) the basal bodies, within an invagination in the chloroplast that fills up the whole posterior half of the cell. The surface of the cell is covered with a cell wall except for two holes through which the flagella protrude. Near the cell surface is an eyespot, which acts as a directional photosensor and allows the cell to swim towards light. The cytoplasmic microtubule cytoskeleton consists of two types of microtubules. Rootlet microtubules are bundles of 2 or 4 microtubules that form a cross-like pattern near the anterior and run down the sides of the cell. The eyespot is always located adjacent to one of these rootlet microtubule bundles. In addition to the rootlet, there is also a set of more conventional singlet microtubules. All of these microtubules, rootlet or not, have their minus ends located near the basal bodies.

### 2.2 *Chlamydomonas* flagella

*Chlamydomonas* flagella are roughly 10 microns long motile structures that are, in all respects, virtually identical to motile cilia in animal cells. They contain the usual nine outer doublet microtubules surrounding a central pair of microtubules. The outer doublets are associated with inner and outer dynein arm complexes as well as radial spokes and the dynein regulatory complex. All of these components share a high degree of molecular homology to their counterparts in other species including humans. On the outside of the flagella are hairlike projects called mastigonemes that are thought to be involved with hydrodynamic interactions (Witman et al., 1972).

The flagella of *Chlamydomonas* were so-named because there are only two of them, not the large number of cilia typically found on ciliates or multi-ciliated epithelial cells. On the other hand, the motility pattern of *Chlamydomonas* flagella is characterized by a breast-stroke like motion of the two flagella in a plane with a recovery stroke near the cell body, a type of motion often referred to as “ciliary.” Moreover, *Chlamydomonas* flagella arise from basal bodies that associate with the poles of the mitotic spindle during division, and by this definition they are classified as primary cilia. There has been some debate in recent years about whether *Chlamydomonas* researchers should switch terminology and start calling the flagella “cilia,” but we do not recommend this because so many of the gene names (see below) refer to the flagella, and in any case the scientific community presumably has the ability to grasp the simple fact that “cilia” and “flagella” are really just two words that have been used to describe the same object, much like “morning star” and “evening star.” Certainly at the level of ultrastructure and biochemical composition, the flagella of *Chlamydomonas* and the motile cilia of humans are virtually indistinguishable.

### 2.3 The basics: how to grow *Chlamydomonas*

One simple advantage of *Chlamydomonas* is that it is extremely easy to grow. *Chlamydomonas* forms colonies on agar plates, which makes isolating clonal lines extremely easy. Although these plates can be grown in temperature controlled, light cycling incubators for precise control of growth conditions, this is not at all necessary, and in most cases they can just be grown on a shelf with a fluorescent light, or even just on the bench top. These colonies on plates are highly robust and can be left for days or weeks without the cells dying. For longer term culture, *Chlamydomonas* can be frozen, and it is in this form that the knockout mutant collection is stored. *Chlamydomonas* can also be grown in liquid cultures, ranging from a few hundred microliters in multiwell plates up to 8L cultures in glass or plastic carboys. Larger cultures are of course possible with bioreactors. The media for growing *Chlamydomonas* is easy to make and costs virtually nothing, since they do not require a carbon source due to their ability to fix carbon from CO_2_ in the air.

Compared to the cost in time and labor of maintaining mammalian cells in culture, *Chlamydomonas* requires essentially no effort, cost essentially nothing, and take almost no time to grow: if you have an idea for an experiment, you can start a liquid culture from a plate or slant and be ready to do your experiment the next day or even later the same day. Growing a large 8L culture for biochemical preparations takes about a week.

### 2.4 Imaging *Chlamydomonas*

Because *Chlamydomonas* flagella protrude from the cell surface, they are easy to see even with student-grade microscopes, provided one can get the cells to hold still. For fixed measurements, cells are simply fixed in glutaraldehyde or Lugol’s iodine, which allow easy visualization and measurement of the flagella. For live imaging, proteins are tagged with GFP or other fluorescent protein tags and expressed as transgenes. Increasingly this tagging is being done at endogenous loci using CRISPR gene editing to insert the tag. Once the fluorescent reporter is expressed, flagella are typically imaged using TIRF microscope (Engel et al., 2009). Because *Chlamydomonas* is a soil alga it has the ability to adhere to, and glide along, solid surfaces such as a glass coverslip. Simply placing a drop of liquid *Chlamydomonas* culture on top of a coverslip is enough for the flagella to adhere, which places the flagella directly in the TIRF field, while the cell body remains outside the TIRF field, allowing protein dynamics within flagella to be imaged at high signal to noise without interference from any protein that may be expressed inside the cell body. The extreme convenience of TIRF imaging in *Chlamydomonas* flagella is another major advantage of this model system, compared to, for example, mammalian cells where the cilia overlay the cell body, and allows transport within the flagellum to be studied quantitatively, at single-molecule resolution (Ludington et al., 2013; Wren et al., 2013; Craft et al., 2015; Chien et al., 2017). *Chlamydomonas* flagella are also well suited for high resolution live cell imaging by label-free methods such as DIC microscopy, due to the fact that the flagellum sticks out on a coverslip away from the cell body, so that there is no wavefront distortion such as what would occur with imaging cilia on the surface of a mammalian cell or inside an animal embryo. DIC imaging provided the first discovery of IFT (Kozminski et al., 1993) and has been used since to measure the speed and frequency of IFT particles (Dentler, 2005).

For electron microscopy, the fact that flagella extend out from the cell surface, can be easily isolated (see below), and adhere to glass surfaces makes it a particularly convenient model organism for ultrastructural studies. The small size of the *Chlamydomonas* cell and its ability to grow in a suspension of individual cells also makes it particularly easy to prepare samples for cryoEM. *Chlamydomonas* continues to be a dominant model organism for cryoEM analysis of ciliary structure, including ultrastructural analyses of dynein arms (Nicastro et al., 2006; Oda et al., 2007; Movassagh et al., 2010; Heuser et al., 2012; Roberts et al., 2012), the dynein regulatory complex (Heuser et al., 2009; Song et al., 2015; Lin et al., 2019; Walton et al., 2023), the central pair apparatus (Hou et al., 2021; Han et al., 2022), radial spokes (Pigino et al., 2011; Barber et al., 2012; Zhu et al., 2019; Poghosyan et al., 2020; Gui et al., 2021; Grossman-Haham et al., 2021), IFT particles (Jordan et al., 2018; van den Hoek et al., 2022; Lacey et al., 2023), and the outer doublet microtubules (Lin et al., 2012; Yanagisawa et al., 2014; Owa et al., 2019; Khalifa et al., 2020).

### 2.5 Isolating *Chlamydomonas* flagella for biochemical analysis

Although we rightfully emphasize the importance of *Chlamydomonas* as a genetic model system for rapid genetic analysis of flagellar processes (see below), it has proven equally valuable for its biochemical tractability. One obvious reason *Chlamydomonas* is so good for biochemistry is just how easy it is to grow in huge quantities. No special bioreactors are required, just a large container, some media which is essentially salt water and costs next to nothing, and a hose to connect the bottle to an air line, with a bubbler or even just a serological pipette attached to one end to introduce the air into the culture in a stream of fine bubbles. House air works for this purpose, there is no need for a CO_2_ line, since the cells can extract what they need from the air. A simple fluorescent light fixture provides plenty of light. It isn’t even necessary to stir the cells: because *Chlamydomonas* are motile, they keep themselves in suspension.

But even more important than the ease and low cost of growing *Chlamydomonas* in biochemical quantities is the fact that *Chlamydomonas* flagella can be induced to detach from the cell using a simple pH shock. The normal pH of a *Chlamydomonas* culture is around 7. By adding HCl, the pH is dropped down to 4.5 for 1 min and then brought back to 7 by adding KOH. During this pH shock, flagella detach from the cell body and float into the media. This takes place without cell lysis, such that once the cell bodies are separated from the flagella by spinning through a sucrose cushion, one obtains a virtually pure sample of flagella, free from contamination with other cellular proteins. The flagella can then be harvested by centrifugation at higher speeds to form a glassy pellet. We are unaware of any other model system that allows cilia and flagella to be isolated in such pure form. In particular, the lack of a method to cleanly remove cilia from mammalian cells in culture has made proteomic studies extremely challenging (see, for example, the mouse primary ciliary proteomic study by Ishikawa et al., 2012, which required hundreds of cell culture dishes and where it was necessary to use comparative proteomics across fractions to identify ciliary proteins by correlation profiling. No such elaborate schemes are required with *Chlamydomonas*).

Given the ability to cleanly isolate flagella with virtually no contamination from the cell body, and the high quality annotation of the *Chlamydomonas* genome, this system is perfect for proteomic analysis of cilia and flagella. The first proteome of the *Chlamydomonas* flagellum (Pazour et al., 2005) was a large advance over the previously published proteomic study of mammalian airway cilia which was full of apparent contamination from other proteins due to the difficulty of isolating cilia without cell damage (Ostrowski et al., 2002). In fairness though, we note that a re-annotation of the mammalian dataset using the better annotated ciliary genes from *Chlamydomonas* showed that quite a few of the proteins found in that study, initially annotated as hypothetical or with uninformative human gene names, were in fact *bona fide* ciliary proteins (Marshall, 2004).

## 3 *Chlamydomonas* genetics

### 3.1 Overview of *Chlamydomonas* genetics

**FIGURE 2:**
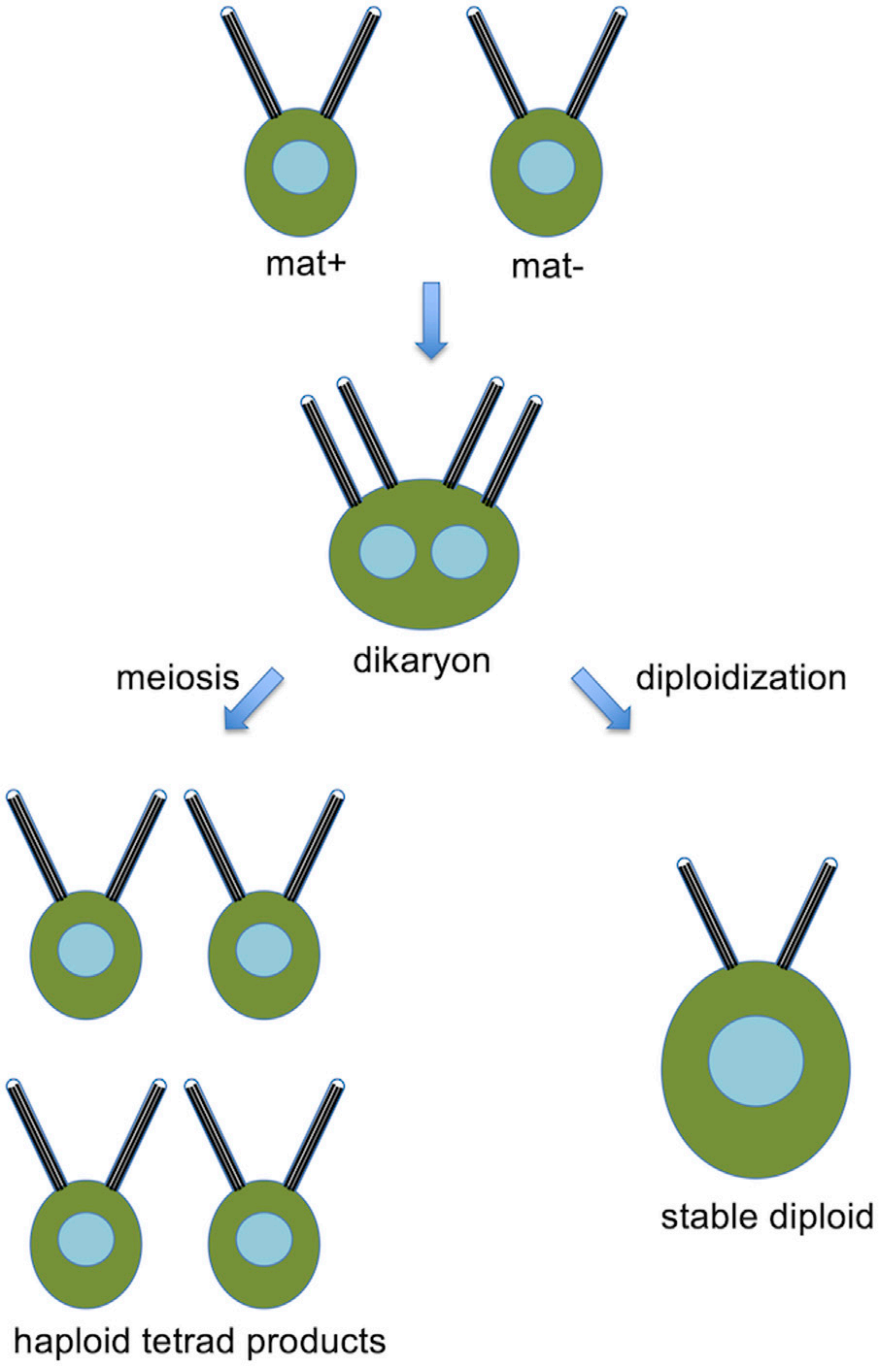
Basic Chlamydomonas genetics, showing a pair of opposite mating type cells that fuse to form a dikaryon, which, can then either undergo meiosis to produce haploid products, or remain as a stable diploid.

*Chlamydomonas* normally grows as a haploid cell with 17 chromosomes per haploid complement. It is heterothallic and exists in two mating types, mat+ and mat-. Haploid cells may be induced to form gametes by growth in nitrogen-free media, and when mat+ and mat-gametes are mixed together, the cells fuse to form a dikaryon with four flagella, which then resorbs its flagella, undergoes meiosis, to yield haploid progeny (Figure 2). A fraction of mated cells remain permanently in the diploid condition.

### 3.2 Methods for genetic analysis in *Chlamydomonas*

Although we often associate tetrad analysis with budding yeast, it is even easier to do in *Chlamydomonas* since the tetrads are larger. The ability to recover all four meiotic products in a tetrad facilitates genetic analysis by making it straightforward to determine if a phenotype segregates 2:2, indicating it is caused by a single gene, and to test for linkage between two mutations based on co-segregation.

By looking at enough tetrads with different markers on known chromosomes, it is straightforward to map the location of a new mutant. One might ask, in the modern era of reverse genetics, knockout collections, and CRISPR editing, why would anyone need to map a mutation? The answer is that all of these methods suffer from occasional off-target mutations, such that even a known and sequenced insertional mutant or gene edit can also be associated with an unknown change elsewhere in the genome. The first step with any experiment involving a new mutant or edited gene is to confirm co-segregation of the insertion or edit with the observed phenotype. In cases where the phenotype can be separated from the insert or edit by a cross, it is then necessary to identify the location of the actual mutation. Genomic sequencing is now the preferred method to do this, but even there, knowing which chromosome is affected can make it much easier to focus on the most relevant SNPs.

The fact that a fraction of cells form diploids allows one to determine if a given mutation is recessive or dominant. Because only a fraction of mated cells will go on to form diploids, they must be selected by using appropriate combinations of markers in the two parental strains.

### 3.3 Dikaryon rescue

The mating process in *Chlamydomonas* also provides a uniquely fast and powerful mechanism for assessing complementation of mutations by cell fusion. When a mat+ and mat-cell mate, the resulting dikaryon retains its flagella (four in total, two from each parental cell) for approximately 2 h. During this time, the cell bodies are fully fused to form one cell, which allows rapid and complete mixing of the cellular contents. If one of the cells had a recessive mutation, the wt protein from the other cell can complement the flagellar phenotype. But this doesn’t always happen, and one can imagine several reasons. First, if the phenotype arises from a defect in a multi-protein assembly, these assemblies may have formed a defective structure in the mutant that does not permit the wild-type protein from the other parent to add on after the structure has assembled. Second, if a gene functions to promote proper flagellar assembly at the time flagella form, it may not be possible to rescue the defect once the flagella are done assembling. Thus, looking at “dikaryon rescue” can shed light on when and how a gene functions, and this approach has been applied to a large number of flagella related mutants (Dutcher, 2014). Formation of dikaryons also provides a convenient way to “pulse label” a cell by introducing a tagged protein from one parent and watching it incorporate into the flagella of the other parent (Marshall and Rosenbaum, 2001).

### 3.4 Screening methods for flagella-related genes

A prerequisite to genetic analysis is a method to screen for mutants of interest. For classical unbiased screening, mutagenesis is carried out by UV or chemical mutagenesis, or by insertional mutagenesis using plasmids. Insertional mutagenesis has the obvious advantage that the mutated gene can, at least in theory, be identified by using the insert as a sequence tag.

After mutagenesis, cells are screened for mutations affecting flagella. Because flagella are completely dispensable for growth and viability, you can’t tell by looking at a colony whether or not the flagella are defective. One approach is to pick individual colonies into liquid cultures and then examine each one under a microscope to see if the cells are swimming. This is a bit tedious but is effective and has the advantage that it is a very direct assay for flagellar motility. Another type of screen takes advantage of the fact that when embedded in a low percentage agar gel, *Chlamydomonas*cells can spread through the gel using flagellar motility, such that a single cell gives rise to a diffuse light green colony. Flagellar mutant cells cannot move through the gel and produce tiny dark green colonies (Engel et al., 2012). Flagellar motility can also be assayed in 96 well plates with U-botton wells. Many strains of *Chlamydomonas* have a tendency to swim downwards to the bottom of whatever container they are placed in, and in a U-bottom well, this means the center of the well. When viewed from above such strains show a dark green dot in the center of each well, while flagellar motility mutants, which lose the ability to concentrate at the lowest point, remain diffuse throughout the well, making them easy to spot. This assay has the advantage of being easy to quantify using image analysis, and has been used for high-throughput chemical screens (see Section 3.8 below).

Another way to identify mutants affecting flagellar motility is to assay phototaxis. When a tube full of liquid *Chlamydomonas* culture is placed in front of a slit of light, the culture will concentrate near the slit, making a green bar when the tube is viewed from the side. This type of screen will pick up mutants defective in flagellar motility, as well as mutants with eyespot defects (see Section 5.3 below).

### 3.5 Cloning by sequencing

Insertional mutants can be cloned by first identifying flanking sequence and then confirming that the insertion is the causal mutation. But many useful mutants have been generated using methods such as UV or chemical mutagenesis, either for historical reasons or with the goal of obtaining conditional mutants that cannot be generated by insertion. Such mutants are now identified by whole genome sequencing to detect alterations in sequence (see, for example, Lin et al., 2013; Perlaza et al., 2022). Because there may be multiple genomic differences in any given mutant strain, it is important to confirm that the proposed gene really is the cause of the phenotype. At the very least, one can perform tetrad analysis to show that the phenotype co-segregates with the genomic change (as verified by PCR, for example,). But the most convincing proof is to perform rescue by showing that insertion of a plasmid carrying the wild-type copy of the gene actually does restore the wild-type phenotype in the mutant line.

### 3.6 Reverse genetics

A large proportion of flagella-related genes in *Chlamydomonas* were first identified by classical “forward” genetics, meaning they were first found by looking for a phenotype of interest in a large collection of randomly mutated cells. In cases where the gene of interest is known, and it is desired to have a mutation in that gene, there are two main strategies currently in use. First, one can simply look up that gene in a database that lists the insertion site sequences in a large knockout collection that is now available to the community (Li et al., 2016). Mutants in this collection generally involve an insertion of a plasmid into the gene, leading to a loss of function. However, the insertion event can sometimes cause additional mutagenic events elsewhere in the genome, so one must always confirm that the observed phenotype results from the insertion, ideally by rescue with a transgene. Now that CRISPR gene editing works well in *Chlamydomonas*, this provides an alternative strategy for obtaining mutants in a gene of interest (Nievergelt et al., 2023) with potentially much greater control over the nature of the mutation compared to random insertional mutagenesis.

### 3.7 Combining genetics and biochemistry

As noted above, *Chlamydomonas* has many advantages for biochemical analysis of flagellar proteins, but the real power comes when these advantages are combined with genetics. Suppose one wants to identify protein components of a particular cilia-associated structure, such as the radial spoke or inner dynein arm. In many cases, mutants are already available that are missing that particular structure while the rest of the flagellum remains intact. One therefore can isolate flagella from wild type cells, and from the mutant in question, and then compare the two samples to identify proteins that are missing in the mutant (Piperno et al., 1977). The clean isolation of flagella that is possible in *Chlamydomonas* makes this type of strategy far more effective in *Chlamydomonas* than in any other ciliary model organism. The large number of well-characterized mutants with defects in specific flagellar structures provides the necessary genetic substrate to support this type of approach. An excellent example of this combined biochemistry/genetics approach has been the identification of the complete protein composition of the radial spoke complex (Curry and Rosenbaum, 1993; Yang et al., 2006).

### 3.8 Chemical screening

In addition to its numerous advantages for genetic screening, *Chlamydomonas* is also highly effective as a model system that allows chemical screening at far lower cost and far higher throughput than other ciliary model systems. A liquid culture of *Chlamydomonas* can be readily dispensed into multi-well plates in a single, rapid step. Motility defects can be assessed in a plate format simply by looking at the distribution of green intensity (signifying accumulation of cells) throughout the well, which can be done using a flatbed scanner or any other simple imaging system (Marshall, 2009; Avasthi and Marshall, 2013). Counter-screening for toxicity is equally simple since it just entails measuring the OD in a well on a plate. Follow-up assays using simple microscopy methods can quickly distinguish effects on flagellar motility from flagellar length. The fact that flagella stick out from the cell body and do not contain obvious drug efflux pumps makes them excellent targets for chemical screens.

This type of approach has been used to discover a number of effective inhibitors of flagellar assembly and motility in *Chlamydomonas* (Engel et al., 2011; Avasthi et al., 2012). Among these compounds are several that were predicted to target a specific class of GPCRs which also turned out to be cilia-related in mammalian cells (Avasthi et al., 2012). These screens also focused interest on actin modulators, which ultimately led to the finding that actin plays a role in transport into the flagellum (Avasthi et al., 2014; Jack et al., 2019; Bigge et al., 2023). For these latter studies, a key element of the approach was using well-characterized genetic mutants in a combined chemical/genetic approach.

## 4 Using *Chlamydomonas* to study flagellar assembly

### 4.1 Flagellar regeneration—a unique advantage of *Chlamydomonas*

**FIGURE 3:**
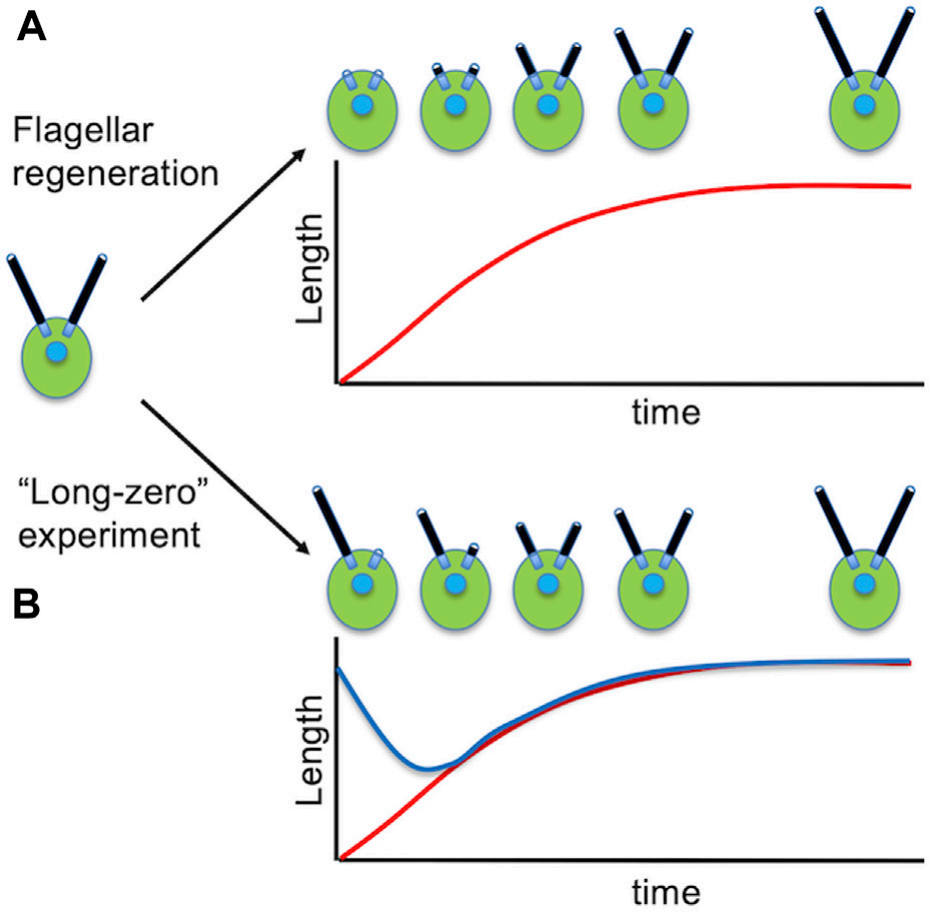
Studying the dynamics of flagellar assembly in Chlamydomonas. **(A)** Regeneration of flagella following pH shock. **(B)** Flagellar length equalization following removal of one flagellum. Red curve shows the length of the severed flagellum as it regenerates. Blue curve shows the length of the other flagellum that shortens until the two flagella reach equal length.

We noted above that a major advantage of *Chlamydomonas* for biochemistry is the ability to cleanly remove the flagella. This turns out to also be a major, and again quite unique, advantage for the study of flagellar assembly (ciliogenesis), because after the flagella are removed, they grow back again in about 1.5 h (Figure 3A). This flagellar regrowth takes place with decelerating kinetics, and the growth rate provides a direct measure of the flagellar assembly process. A flagellar regeneration curve allows not only the initial growth rate, but also the final length, to be easily determined. Because protein synthesis is required for flagella to reach the final correct length (Rosenbaum et al., 1969), these experiments can also be used to measure the effective size of the flagellar precursor pool (Lefebvre et al., 1978).

### 4.2 The BLD and FLA mutants

Two main classes of mutants have been identified in *Chlamydomonas* with severe effects on flagellar assembly. Both types of mutants are characterized by lack of flagella, the difference is whether the mutations are conditional or not.

One class of mutants comprises non-conditional mutants lack flagella entirely, and were thus named bld and define genes necessary for building either the basal bodies or the flagella. Examples of BLD genes that have been characterized include BLD1, which encodes a protein necessary for intraflagellar transport (Brazelton et al., 2001; see below), BLD2, which encodes epsilon tubulin (Dutcher et al., 2002), BLD10 which encodes part of the basal body cartwheel structure (Matsuura et al., 2004), and BLD12 which encodes the SAS6 centriole assembly protein (Nakazawa et al., 2007). These mutants have proven highly useful in exploring the mechanisms for establishing 9-fold symmetry in centriole structures (Hiraki et al., 2007; Nakazawa et al., 2007; Noga et al., 2022).

A second class of mutants affecting flagella are conditional mutants, known as “FLA” for flagellar assembly defective, in which cells have normal or approximately normal length flagella when grown at the permissive temperature, but are unable to regenerate flagella when the temperature is increased to the nonpermissive temperature (Huang et al., 1977; Adams et al., 1982). Many of these mutants also lose their pre-existing flagella when the temperature is shifted. Examples of FLA genes that have been characterized include FLA3, FLA8, and FLA10 which encode the three subunits of a heterotrimeric kinesin involved in anterograde intraflagellar transport (Walther et al., 1994; Miller et al., 2005; Mueller et al., 2005), and FLA14 which encodes a light chain of the dynein complex responsible for retrograde IFT (Pazour et al., 1998). The fact that flagella shorten when IFT is abruptly halted in such mutants was the first indication that flagella were dynamic rather than static assemblies, and led to a concept for length control based on a competition between assembly and disassembly as discussed below in Section 6.4. An interesting feature of many of these fla mutants is the fact that when IFT is impaired by the mutation, it leads not only to flagellar shortening, but also to severing of the flagella (Parker and Quarmby, 2003).

### 4.3 IFT in *Chlamydomonas*

Intraflagellar transport (IFT) was first discovered as a motility visible within *Chlamydomonas* flagella by DIC microscopy (Kozminski et al., 1993). Building on the observation that IFT stops in fla10 mutants when they are shifted to the restrictive temperature (Kozminski et al., 1995), comparison of proteins in flagella isolated from wt and fla10 mutant flagella revealed a set of polypeptides that were named the IFT proteins (Piperno and Mead, 1997; Cole et al., 1998). Determining the molecular identity of the IFT proteins (Cole et al., 1998) was the key observation that launched the renaissance of interest in cilia after it was recognized that one of the IFT proteins from *Chlamydomonas* was an ortholog of a gene, of previously unknown function, implicated in polycystic kidney disease (Pazour et al., 2000). Due to the combination of advantages for taking genetic, biochemical, and imaging approaches in *Chlamydomonas*, IFT has become extremely well characterized in this organism, including numerous studies of the structure (Pigino et al., 2009; Vannuccini et al., 2016; Jordan et al., 2018; Lacey et al., 2023) and biochemical composition (Cole et al., 1998; Lucker et al., 2005; Qin et al., 2007; Taschner et al., 2011; Behal et al., 2012; Taschner et al., 2016) of the IFT particles, their movements back and forth in the flagellum (Pedersen et al., 2005; Ludington et al., 2013; Chien et al., 2017), their recruitment to the basal body (Deane et al., 2001; Richey and Qin, 2012; van den Hoek et al., 2022), and their transport of cargo (Qin et al., 2004; Bhogaraju et al., 2013; Wren et al., 2013; Craft et al., 2015; Jiang et al., 2017; Taschner et al., 2017; Perlaza et al., 2022). Overall, it is fair to say that we know more about the details of IFT in *Chlamydomonas* than in any other model organism, making it a logical starting point for future investigations into this process.

### 4.4 Gene expression during flagellar assembly

The ability to remove flagella and have them regenerate synchronously in a population of cells makes it easy to study gene expression during flagellar growth, something far more difficult to analyze in organisms where ciliogenesis occurs asynchronously. When flagella regenerate in *Chlamydomonas*, genes encoding virtually all flagellar proteins are upregulated, showing increased levels of mRNA peaking around 20–30 min after the start of flagellar assembly (Lefebvre et al., 1980; Baker et al., 1984; Keller et al., 1984; Schloss et al., 1984; Lefebvre and Rosenbaum, 1986). This phenomenon has been extremely useful in identifying flagella-related genes based on transcriptomic analysis (Pazour et al., 2005; Stolc et al., 2005; Chamberlain et al., 2008; Albee et al., 2013; Lin and Dutcher, 2015; Zones et al., 2015).

How this gene regulation occurs is still very much an open question. It has been shown that flagellar gene induction requires not just loss of flagella, but their ability to regrow, such that mutations that block or delay flagellar regeneration greatly reduces gene induction (Perlaza et al., 2023). Not just removal of flagella, but also induction of further growth of flagella, is sufficient to trigger flagella related gene expression (Periz et al., 2007). The molecular basis by which flagellar growth is coupled to gene expression is currently unknown. Mutations in the promotor region of flagella related genes can prevent their upregulation during flagellar assembly (Davis and Grossman, 1994), but the factors necessary to bind to promotor sequences in response to flagellar growth are not known. A transcription factor has been identified, XAP5, that is required for cells to have flagella (Li et al., 2018), but because the presence of flagella and their subsequent regeneration is required for flagellar gene induction (Perlaza et al., 2023), it is not possible to test whether this particular factor is a necessary part of the normal induction machinery. While the question of how growth of cilia and flagella are coupled to gene expression is a very general one, *Chlamydomonas* has huge advantages for exploring the molecular mechanism due to the ability to induce flagellar regeneration precisely and synchronously, on top of the usual advantages in terms of being able to carry out rapid screens in haploid cells.

## 5 Using *Chlamydomonas* to study flagella-based motility

### 5.1 Motility mutants

Three classes of paralyzed flagella mutants have been reported: pf, ida, and oda (Kamiya, 1988). PF refers to “paralyzed flagella” and was the original term used to name genes, mutations in which cause defects in swimming motility. Over the years, the identity of many of the original PF genes have been determined. In some cases, these have been identified directly by either genetic mapping followed by complementation or by use of insertional mutants from which the flanking DNA can be determined. In addition, a significant number of paralyzed mutants showed loss of specific structures by electron microscopy, such that analysis of proteins lacking from mutant flagella pointed the way to potential protein products of the mutated genes.

Flagellar motility ultimately is produced by the inner and outer dynein arms, and given the complexity of the dynein arms, each of which is composed of many different chains, it is not surprising that a large number of motility mutants turn out to affect dynein arm assembly or function. Axonemal dynein arms in *Chlamydomonas* pre-assemble in the cytoplasm and are then transported into the flagellum and attached to the axoneme (Fowkes and Mitchell, 1998). While some of the ida and oda mutations affect genes encoding components of the dynein arms themselves (as just a few examples see Kamiya et al., 1991; Sakakibara et al., 1993; Pazour et al., 1999; Perrone et al., 2000), many affect genes required for the assembly, transport, or docking of the arms onto the axoneme (Wirschell et al., 2004; Ahmed and Mitchell, 2005; Oda et al., 2014). Many, but not all, of the ida and oda mutants, show short flagellar length (Kannegaard et al., 2014) possibly because their binding to the outer doublet serves to stabilize it against turnover (see Section 6 below).

The activity of inner dynein arms is coordinated in part by signaling through the dynein regulatory complex (DRC; Piperno et al., 1992; Gardner et al., 1994). This protein complex turns out to be the “nexin” link observed decades earlier by electron microscopy (Heuser et al., 2009). Mutations in the DRC lead to alterations in swimming motility (Piperno et al., 1994). Adjacent to the DRC on the outer doublets and pointing towards the central pair, are the radial spoke complexes (Curry and Rosenbaum, 1993). Like the dynein arms, radial spokes are partially assembled in the cytoplasm and then transported to the axoneme for final attachment (Diener et al., 2011). Mutations in components of the radial spokes, or the central pair itself, lead to paralyzed flagella or impaired motility (Williams et al., 1989; Smith and Lefebvre, 1996; Mitchell and Sale, 1999).

### 5.2 Gliding motility

In addition to the canonical “swimming” motility that involves bending of the flagella, *Chlamydomonas* also can undergo a “gliding” motility, whereby the retrograde IFT motor system engages with membrane proteins (Bloodgood et al., 2019) that adhere to an external surface, causing the cell body to be dragged forward (Fort et al., 2021). One reason to investigate gliding is that it may provide a window into the evolutionary origins of cilia. Canonical flagellar or ciliary motility requires a huge number of components to interact in the correct way, but surface motility like gliding is far simpler to achieve, can be used to capture prey (Bloodgood, 2020) or possibly to avoid predation (Marshall, 2020a). Such considerations have led to speculation that cilia may have evolved from structurally simpler precursors whose main function was surface motility of the membrane. *Chlamydomonas* gliding provides a genetically tractable system in which to investigate the mechanism of such processes.

A related surface motility process, likely reflecting the same underlying machinery, is the motion of adherent beads back and forth over the flagellar surface (Bloodgood, 1977). Such bead movement is convenient to measure and possibly relates to early evolution origins of cilia-like structures as feeding organelles.

### 5.3 Phototaxis

Phototaxis in *Chlamydomonas* is accomplished by a complex interaction between the eyespot, which is a directional photodetector formed by rhodopsin-like photoreceptors overlying a pigment layer that blocks light from one side, and flagellar motility, such that when light impinges on the eyespot, the motility of one of the two flagella is altered in such a way that the cell swims towards the light (Wakabayashi et al., 2021). Because of the ease with which phototaxis mutants can be identified in screens, this system presents an excellent opportunity to investigate the general question of how ciliary motility can be controlled by cellular signaling pathways, which is relatively much harder to investigate in other model systems.

Because phototaxis requires an interaction between the eyespot and flagellum, mutations that disrupt phototaxis can arise from defects in the flagellum, the eyespot, or the signaling pathway that links them. Phototaxis relies in the fact that the two flagella respond differently to light, which allows the cell to turn. The difference in response between the two flagella ultimately depends on which basal body (mother or daughter) served to template the assembly of the flagellum. Mutations that affect the asymmetry between the two flagella lead to loss of phototaxis (Rüffer and Nultsch, 1997; Okia et al., 2005).

Mutants affecting eyespot formation also lead to impaired phototaxis, and include mutants with miniature eyespots, multiple eyespots, and completely missing eyespots (Mittelmeier et al., 2008; Boyd et al., 2011a) as well as mutants with eyespots in an incorrect location due to alterations in rootlet microtubules (Boyd et al., 2011b). Control of flagellar beating by the eyespot involves not only ion current through the eyespot itself, but also a calcium channel in the flagellar membrane, such that mutants affecting signaling can arise from defects in flagellar calcium influx (Pazour et al., 1995). Effective phototaxis also requires a precise angular position of the eyespot relative to the flagella, such that mutants affecting eyespot or basal body positioning lead to loss of phototaxis (Feldman et al., 2007).

## 6 Using *Chlamydomonas* to study flagellar length regulation

### 6.1 Do cells “know” how long their flagella are?

Cilia and flagella are convenient organelles in which to investigate the general question of how cells regulate organelle size (Marshall, 2020b). Advantages of cilia/flagella for this question include the fact that their size only varies in one dimension (length), their large size on the order of several microns, and the fact that they typically protrude from the surface of the cells making them easy to visualize. Moreover, unlike many organelles, flagella in *Chlamydomonas* can be removed from the cell and induced to regenerate in a highly synchronous manner, allowing the kinetics of length restoration to be measured. *Chlamydomonas* flagella thus represent a highly tractable system in which to investigate the general question of organelle size control using a combination of yeast-like genetics and precise quantitative measurements (Wemmer and Marshall, 2007).

Flagellar length is usually around 10 microns but can vary from 8 to 12 in wild-type strains depending on the exact strain and growth conditions. Length also is a function of cell size, with larger cells having longer flagella, showing a linear scaling of flagellar length with cell diameter (Bauer et al., 2021). Flagellar length is important for swimming motility, such that cells with lengths outside the normal range, or with unequal flagellar lengths, show impaired swimming (Bauer et al., 2021).

A basic question is whether flagellar length is actually under any sort of regulatory control. When flagella regenerate, they return to their original length, and do so with decelerating kinetics such that at any point in time, the growth rate is proportional to 1/L (Marshall et al., 2005). When fluctuations of flagellar length are measured, we find that lengths can increase and decrease randomly but only within a limited range of variation, beyond which they are somehow prevented from growing or shrinking (Bauer et al., 2021). The restoration of length following perturbation, and the constrained fluctuations about the mean length, are both hallmarks of a control system regulating length. Another reason for thinking that the cell contains signaling pathways that can measure length is the fact that some proteins show length-dependent phosphorylation (Luo et al., 2011).

### 6.2 Flagellar length equalization

One of the most compelling reasons for suspecting that some mechanism exists to regulate flagellar length in *Chlamydomonas* is the fact that cells are able to restore equal lengths of their flagella. This is seen most dramatically in the so-called “long zero” experiment, in which a single flagellum is removed, either by careful mechanical perturbation (Coyne and Rosenbaum, 1970) or more recently by laser ablation of just one flagellum (Ludington et al., 2012). In these experiments, the flagellum that is removed regenerates with similar kinetics as seen when both flagella are removed. The remarkable thing, however, is that the other flagellum, which was at normal length, immediately starts to shorten, and continues shortening until it matches the length of the regenerating flagella (Figure 3B). Once they reach the same length, the two flagella grow out together, unless protein synthesis is inhibited, in which case the two flagella reach equal length and then stop further growth.

### 6.3 Mutants affecting flagellar length

A principal reason for studying flagellar length in *Chlamydomonas* is the genetics of the system. Using screens, primarily for alterations in motility which is an easily scored phenotype, a number of length-altering mutants have been identified, which fall into two main classes: long-flagella (lf) mutants with flagella typically around twice normal length (Barsel et al., 1998; Berman et al., 2003; Tam et al., 2003; Tam et al., 2007; Tam et al., 2013), and short flagella (shf) mutants with flagella around half normal length, still long enough to be clearly visible but outside the range of wt flagellar lengths (Mcvittie, 1972; Kuchka and Jarvik, 1987; Perlaza et al., 2022).

Five of the LF genes have been identified (Berman et al., 2003; Nguyen et al., 2005; Tam et al., 2007; Tam et al., 2013). Three of them, LF2, LF4, and LF5, encode protein kinases, but their targets or upstream regulators remain poorly understood. LF4 seems to be involved in phosphorylating a subunit of the kinesin that drives IFT (Wang et al., 2019), which based on other studies (Liang et al., 2018) is predicted to decrease IFT entry into flagella. This leads to a simple model in which mutation of LF4 leads to reduced kinesin phosphorylation and increased IFT. Indeed, LF4, as well as LF1 and 2, appears to be involved in the regulation of IFT based on quantitative measurement of IFT in mutant cells (Ludington et al., 2013; Wemmer et al., 2020). LF4 is not, however, necessary for length control: lf4 null mutants still grow flagella to defined lengths, the defect is just that the length set-point is longer than in wild-type cells.

Compared to long flagella mutants there has been comparatively less effort to identify the genes altered in short flagella (shf) mutants. This is due to the perception, not usually stated explicitly, that there are more ways to make something smaller than to make it larger. On the other hand, the fact that shf mutants have defined lengths that are different from wt cells still raises the question of what has changed in such cases. The first short flagella gene identified, sfh1, was recently shown to encode a TOG-domain tubulin binding protein, suggesting a role in tubulin transport into flagella (Perlaza et al., 2022). A short flagella mutant obtained in a screen for phototaxis defects (Feldman et al., 2007) was found to be caused by a mutation in the PF15 gene encoding the p80 regulatory subunit of katanin (Kannegaard et al., 2014). Since katanin in *Chlamydomonas* seems to be localized in the cytoplasm near the basal bodies rather than inside the flagella (Esparza et al., 2013), this result suggested that perhaps katanin plays a role in destabilizing cytoplasmic microtubules during flagellar assembly, allowing the growing flagella to compete for the limited pool of tubulin, an idea that was supported by computational modeling (Kannegaard et al., 2014).

### 6.4 Turnover and transport

Although identifying the genes in flagellar length altering mutants has represented a major point of progress in understanding length regulation, it does not by itself point to an actual mechanism for controlling length. A key question for any mechanistic model for organelle size regulation is to know whether the control is exerted only at the time of initial assembly, or continuously throughout the life of the organelle. In the case of *Chlamydomonas* flagella, the answer is clearly the latter: flagellar outer doublet microtubules undergo continuous turnover at their tip (Marshall and Rosenbaum, 2001; Song and Dentler, 2001) and immediately begin to shorten when IFT is shut off (Kozminski et al., 1995). The fact that shortening in the absence of IFT occurs at a constant rate shows that microtubule disassembly occurs at a length-independent rate. Since assembly requires IFT, we have extensively investigated the rate of IFT as function of length and find that the rate of IFT particles leaving the base of the flagellum to travel out to the tip is proportional to 1/L, just like the rate of flagellar assembly itself (Engel et al., 2009; Ludington et al., 2013). Interestingly, the size of IFT trains changes as a function of flagellar length, with much longer trains being found in short, rapidly growing flagella (Engel et al., 2009). Mutations that partially reduce IFT lead to shorter steady state flagellar lengths (Marshall and Rosenbaum, 2001), and mutations that increase IFT lead to longer steady state flagellar length (Wemmer et al., 2020).

**FIGURE 4:**
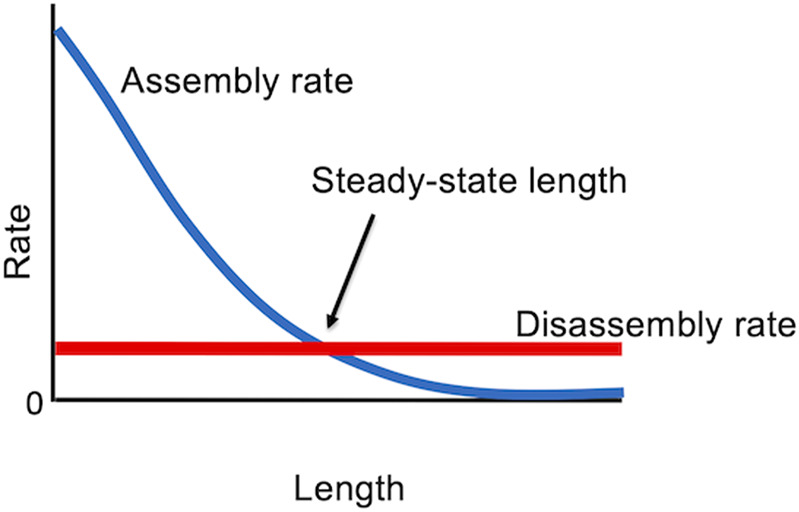
Steady state “balance point” model for flagellar length, in which the only stable length occurs when the rate of assembly matches the rate of disassembly.

With these results in hand, it is possible to formulate an extremely simple model for flagellar length control (Marshall and Rosenbaum, 2001; Marshall et al., 2005), in which the steady-state flagellar length is determined by the balance between length-dependent assembly (due to the 1/L dependence of IFT on length) and length-independent disassembly (Figure 4). This model can explain the ability of cells to stably maintain a unique length, as well as the regeneration kinetics of flagella as they regrow, since the growth rate turns out to show the same 1/L dependence on length as does IFT (Marshall et al., 2005). The model can also account for length equalization in the “long-zero experiment” because when one flagellum is removed, and starts to regrow, it creates a situation in which that flagellum is taking up more tubulin than it returns by disassembly, so it becomes a net consumer, thus reducing the supply available for the other flagellum (Ludington et al., 2012). The model also explains the fact that in cells with variable numbers of flagella, the more flagella a cell has, the shorter they are (Marshall et al., 2005).

Here we have discussed only the simplest version of the model. Building in some of the known or potential complexity of the system, such as length-dependence of disassembly, saturable binding of tubulin to IFT particles, and limited availability of precursor proteins, does not fundamentally alter the ability of this type of model to account for length equalization and the ability to reach a stable steady state length (Marshall, 2023).

### 6.5 Models for IFT regulation

The length control model discussed in the last section only works if IFT is a decreasing function of length, which raises the question of how this can be achieved? There are a large number of potential mechanisms by which the length of the flagellum could be sensed, many of which have been shown to be capable, in theory, of fitting available measurements of flagellar regeneration (Ludington et al., 2015). Testing these models has employed a combination of specific mutations with quantitative imaging, which has allowed us to rule out three models so far: (a) an “initial bolus” model where flagella are loaded with a fixed quantity of IFT particles that circulate back and forth, ruled out by photobleaching experiments (Ludington et al., 2015); (b) a “time of flight” model where length is sensed by a molecular timer attached to the IFT particle, ruled out using mutants that change the speed of retrograde IFT (Ishikawa and Marshall, 2017); and (c) an “ion current” model where length is sensed by the magnitude of a calcium current flowing across the flagellar membrane, ruled out using mutations and chemical perturbations that alter flagellar calcium transport (Ishikawa et al., 2023).

One current model is based on the observation that the KAP subunit of the heterotrimeric kinesin-II driving anterograde IFT does not return to the cell body by retrograde IFT, but instead, diffuses back in a random walk (Chien et al., 2017). This led to a model in which the entry of new IFT trains is delayed until sufficient kinesin has been able to return by diffusion, and this model was shown to be able to fit with the observed kinetic data using the measured diffusion constant (Hendel et al., 2018; Ma et al., 2020). When a kinesin-II motor with reduced speed was expressed in *Chlamydomonas*, this had only a small effect on steady state length, consistent with the idea that the timescale of kinesin diffusion, rather than its anterograde motion out to the tip, is limiting (Li et al., 2020). A recent study confirmed the diffusive return of kinesin by tracking the Fla8 subunit (Patel et al., 2024), but reported that the diffusing pool of kinesin is only a small fraction of the total available kinesin, raising questions about the ability of a diffusion model to explain the length dependence of IFT.

### 6.6 Regulating cargo loading and precursor availability

In addition to the length-dependence of IFT, quantitative imaging also suggests that the loading of tubulin and other cargos onto the IFT trains may be regulated by flagellar length (Wren et al., 2013; Craft et al., 2015; Patel et al., 2024). Thus far, no mechanism has been proposed by which this loading would be regulated by length, and so cargo loading is not in itself a solution to the question of how length is sensed. It has also been noted that because the number of IFT particles per IFT train is itself length dependent (Engel et al., 2009), the overall capacity of an IFT train to transport tubulin or other cargos will vary with length simply by virtue of the fact that the number of binding sites is length-dependent (Wemmer et al., 2020). Clearly, the jury is still out on whether IFT or its cargo binding are the primary targets for length regulation, and in either case, what sort of molecular pathway could be sensing length in the first place. The fact that we can even entertain such a debate at this level of detail is a testimony to the numerous advantages of *Chlamydomonas* including not only its genetics but also the ability to induce flagellar regeneration and the ability to acquire TIRF images of IFT at extremely high signal to noise ratios, all of which continue to make this system the idea model organism in which to investigate the general question of ciliary length control.

## 7 Discussion: *Chlamydomonas*—why use anything else?

In this article we have seen the numerous practical advantages that make *Chlamydomonas* such an excellent system for studying cilia and flagella. For virtually any question that applies to cilia in general, such as how they assemble, how they move, and what they are made of, there is simply no question that *Chlamydomonas* allows such fundamental questions to be answered more easily, more quickly, and with greater genetic precision. The questions for which *Chlamydomonas* is not as useful are those that apply only in a multicellular context, such as the roles of cilia in hedgehog signaling or kidney homeostasis. Even there, there are likely to be aspects of the system for which the awesome power of *Chlamydomonas* genetics and biochemistry can be brought to bear. For example, while *Chlamydomonas* doesn’t have a kidney, it has an ortholog of PKD2 (Huang et al., 2007) and recently it has been shown that the mastigoneme protein that forms filaments on the outside of the *Chlamydomonas* flagellum (Witman et al., 1972), is a structural homolog of PKD1 (Huang et al., 2024), and interacts with PKD2 (Das et al., 2024), suggesting that *Chlamydomonas* really does have something equivalent to the PKD1/2 system in mammalian kidneys. In fact, a startlingly large number of genes involved in human ciliopathies have orthologs in *Chlamydomonas*, consistent with the high conservation of ciliary proteins. We conclude that for both basic cell biology of cilia, as well as for more specialized disease-related functions. *Chlamydomonas* has a unique role to play whenever one wants to study such process using yeast-like genetics, combined genetic and biochemical analysis, and high resolution quantitative live cell imaging.
